# Approaching environmental human thermophysiological thresholds for the case of Ankara, Turkey

**DOI:** 10.1007/s00704-020-03436-5

**Published:** 2020-10-27

**Authors:** A. Santos Nouri, Y. Afacan, O. Çalışkan, Tzu-Ping Lin, A. Matzarakis

**Affiliations:** 1 Department of Interior Architecture and Environmental Design, Faculty of Art, Design and Architecture, Bilkent University, 06800 Bilkent, Turkey; 2 Department of Turkish and Social Sciences Education, Faculty of Educational Sciences, Ankara University, Cebeci, Ankara, Turkey; 3 Department of Architecture, National Cheng Kung University, 1 University Rd, East Dist., Tainan, 701 Taiwan; 4 Research Centre Human Biometeorology, German Meteorological Service, 79104 Freiburg, Germany; 5 Environmental Meteorology, Faculty of Environment and Natural Resources, Albert-Ludwigs-University, 79085 Freiburg im Breisgau, Germany

**Keywords:** Human energy balance, Thermal comfort, PET, mPET, UTCI, Ankara

## Abstract

The disclosed study undertook a ‘human centred-approach’ that ascertained and categorised environmental human thermophysiological risk factors by relating them to the human biometeorological system through the use of three widely utilised energy balance model (EBM) indices, the physiologically equivalent temperature (PET), the modified PET, and the universal thermal climate index (UTCI). The disclosed assessment was carried out over the past decade (i.e., 2010–2019) with a 3-h temporal resolution for the case of Ankara through two WMO meteorological stations to compare both local urban and peri-urban environmental conditions. The study recognised extreme annual variability of human physiological stress (PS) during the different seasons as a result of the biometeorological processing of the singular variables, which in the case of average PET for both stations, varied by up to 75 °C between the winter and summer for the same annual dataset (2012). In addition, all EBMs indicated higher heat stress within the city centre that were conducive of both urban extreme heatwaves and very hot days during the summer months, with extreme heat stress levels lasting for longer than a week with PET values reaching a maximum of 48 °C. Similar cold extremes were found for the winter months, with PET values reaching − 30 °C, and average PS levels varying lower in the case of the peri-urban station.

Graphical abstract
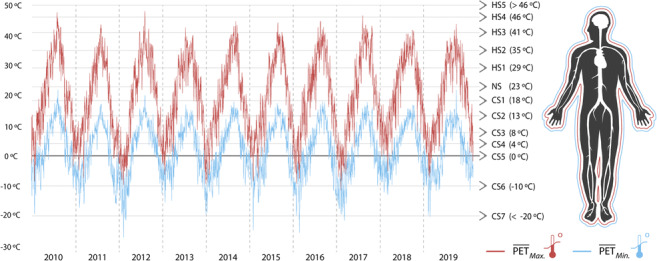

## Introduction

When considering the existing literature of thermal comfort studies thus far, there has been a promising expansion of studies which aim to approach, examine, and improve thermophysiological conditions within urban environments. Such efforts can arguably be associated to the growing climate change adaptation agenda that has further instigated the scientific community to improve the overall climatic responsiveness towards both indoor and outdoor contexts. As the growing need for applicative interdisciplinary knowhow augments amongst different disciplines, the multifaceted topic of environmental biometeorology is continually growing in importance in light of long-term human wellbeing, safety, and prosperity (Alcoforado 1996; Christen 2020; Ketterer and Matzarakis 2014; Nouri and Matzarakis 2019; Shashua-Bar et al. 2012; Vanos et al. 2010). Promisingly, and even in very novel global issues, such interdisciplinary application in environmental scopes continues to be espoused, including to the SARS-CoV-2 pandemic as exemplified by the recent review study undertaken by Cheval et al. (2020).

Ultimately, such a ‘human-centred approach’ relays to the aim in identifying and adjusting local microclimatic factors in order to ensure thermal responsive urban environments (Hebbert and Mackillop 2011; Reiter and Herde 2003; Wilbanks and Kates 1999). Respectively, and while both global circulation models (GCMs) and regional climate models (RCMs) shall continue to play an indispensible scientific role in understanding more encompassing climatic dynamics, an equally major consideration must be made to the relationship of such direct dynamics upon the human biometeorological system. As a result, and in alliance with such top-down approaches, bottom-up approaches must play a pivotal role in comprehending the crucial dynamic relationship between bioclimatic stimuli upon the human body. Moreover, although tackling the exposures of cities to hazards that have a huge impact but low frequency is fundamental, the reversal of this paradigm is also valid. In other words, and as recognised early on, ensuring the management of the ongoing high-frequency microscale climatic stimuli within the anthropogenic environment is just as crucial (Hebbert and Webb 2007; Höppe 1984; Höppe 1999; Oke 1988; Olgyay 1963).

In line with numerous recent studies, which maintain such perspectives (e.g., Binarti et al. 2020; Charalampopoulos 2019; Coccolo et al. 2016; Miao et al. 2019; Nouri et al. 2018a; Potchter et al. 2018; Yang and Matzarakis 2019), it is recognised that the ‘human-centred approach’ requires the use of a thermal comfort indices in order to wholesomely evaluate urban thermophysiological conditions. As identified by studies undertaken by Freitas and Grigorieva (2015), Freitas and Grigorieva (2016), and Spagnolo and de-Dear (2003), since the turn of the century, over 160 human thermal comfort indices have been elaborated to assess thermal stress conditions. From this vast amount of samples, and in accordance with posterior review studies, including those undertaken by Potchter et al. (2018) and Staiger et al. (2019), only a very limited number of these are recurrently utilised to evaluate human biometeorological responses to urban environmental conditions.

Considering the Turkish capital city of Ankara, where few thermophysiological studies have thus far been undertaken, this study analyses the physiologically equivalent temperature (PET) (Höppe 1999; Mayer and Höppe 1987), the modified PET (mPET) (Chen and Matzarakis 2017), and the universal thermal climate index (UTCI) (Bröde et al. 2012; Jendritzky et al. 2012; Jendritzky et al. 2002) for the last decade. Such indices were calculated through the collection and processing of singular climatic variables from two meteorological stations (MSs) through the use of the biometeorological model RayMan Pro© (Matzarakis and Rutz 2006; Matzarakis et al. 2010). Attentive to the ‘human-centred approach’, the study launches a pilot investigation into three interconnected aspects that identifies the (i) behavioural differences between the three energy balance model (EBM) indices; (ii) variances in thermophysiological conditions based on the data provided by two WMO MSs located in different locations in Ankara; and, lastly; (iii) overall human physiological stress (PS) within the city to obtain an encompassing comprehension of the annual/seasonal/diurnal periodicity and intensity in terms of both heat and cold levels.

## Material and Methods

### Köppen-Geiger classification and threshold identification

According to Peel et al. (2007), the principal Köppen-Geiger (KG) classification of Ankara is that of ‘*Dsb*’ which equates to a cold climate, with warm and dry summers (Table 1). At a greater scale however, and resultant of being surrounded by three different seas, sizeable mountain ranges, and interior basins, Turkey witnesses a wide range of climatic classifications. In general, and in alignment also with the posterior KG map produced by Rubel and Kottek (2010), the country ranges between the ‘B’, ‘C’, ‘D’, and even ‘E’ classifications, with a further extensive variation between their sub-classifications. To produce a more detailed analysis of such a disparity of climatic classifications, and based on meteorological station data as well, Yılmaz and Çiçek (2018) produced newer maps of regional KG distribution for Turkey. In the case of its capital, the aforementioned study demonstrated that while plateau areas within the central and eastern Anatolian regions presented a ‘*Dsb*’ classification, this however varied to ‘*Dsa*’ within the depressions of the Anatolian plateaus. Furthermore, it was also identified that in the particular case Ankara, it was additionally adjacent to further classifications, namely that of ‘*Csa*’ and ‘*BSk*’. In parallel, and also focusing upon the case of Turkey, building upon the earlier outputs obtained by Peel et al. (2007), such variations in KG in adjacent areas of Ankara were also identified by Öztürk et al. (2017), including the classification of ‘*BSk*’ as well.

**Table 1:** Descriptive environmental summary of the Köppen-Geiger classification system according to Peel et al. (2007)

KG class.	Colloquial name of sub-classification		Specific environmental thresholds			
		General classification descriptors	Precipitation descriptors		Temperature descriptors	
			General description	Climate specification	General description	Climate specification
‘*Dsb*’	Snow/cold climate with dry/warm summer	T_hot_ ≤ 21 °C and T_cold_ ≤ 0	Dry summer	P_sdry_ < 40 and P_sdry_ < P_wwet_/3	Warm summer	T_hot_ ≤ 21 °C and T_mon10_ ≥ 4
‘*Dsa*’	Snow/cold climate with dry hot summer	T_hot_ ≤ 21 °C and T_cold_ ≤ 0	Dry summer	P_sdry_ < 40 and P_sdry_ < P_wwet_/3	Hot summer	T_hot_ ≥ 22 °C
‘*Csa*’	Warm temperate with dry hot summer	T_hot_ > 10 °C and T_cold_ < 18	Dry summer	P_sdry_ < 40 and P_sdry_ < P_wwet_/3	Hot summer	T_hot_ ≥ 22 °C
‘*BSk*’	Cold semi-arid climate	MAP < 10 × P_threshold_	Steppe	MAP ≥5 × P_threshold_	Cold	MAT <18 °C

*MAT*, mean annual temperature; *T*_*hot*_, temperature of the hottest month; *T*_*cold*_, temperature of the coldest month; *T*_*mon10*_, number of months where the temperature is above 10; *MAP*, mean annual precipitation; *P*_*sdry*_, precipitation of the driest month in summer; *P*_*wdry*_, precipitation of the driest month in summer; *P*_*wwet*_, precipitation of the wettest month in winter; *P*_*threshold*_, 2 × MAT

As can be seen from variability presented in Table 1, it is possible to verify that the case of Ankara, and its immediate surrounding areas, has raised some incongruity in terms of its KG classification. As suggested by existing literature, such climatic classification ‘zone traversing’ in Turkey can be attributed to numerous reasons, including (i) subjectivity in classification stipulation rules (Unal et al. 2003), and (ii) local precision as a result of lacking data, and scaling issues (particularly in light of Turkey’s geographical and topographical characteristics) (Yılmaz and Çiçek 2018). While both valid explanations, the weight of the latter is arguably more befitting.

Based upon two singular variables, air temperature (T_a_) and precipitation (P_R_) data, the KG classification remains one of the most utilised categorisation systems, and is extensively employed within climatic studies (Chen and Chen 2013). Albeit an effective top-down means to initially determine/comprehend environmental conditions in different regions, when returning to the ‘human-centred approach’ utilised in this study, the KG system proves insufficient. This can be relayed to two interconnected explanations, namely (i) as reiterated by Yang and Matzarakis (2016), while T_a_ and P_R_ variables are capable of efficiently determining hot/cold and wet/dry conditions, the KG system is designed to determine the effects upon vegetation (as ascertained by Rubel and Kottek (2011)) rather than concretely upon the human biometeorological system; and in association, (ii) the lack of consideration of other singular variables does not permit the encompassing thermophysiological understanding of how the human body shall react under such environmental conditions under a type of KG classification (Djamila and Yong 2016; Nouri and Matzarakis 2020; Yang and Matzarakis 2019).

### Selection of singular climatic variables

Within this study, six singular climatic variables over the past decade from two selected MSs were obtained, and later processed into the three EBM indices. Equating to a total of eight recordings, the variables were retrieved at a 3-h interval, enabling the understanding of their diurnal/nocturnal fluctuations between the years of 2010 and 2019. The selection of these variables is attributed to their importance in determining the physiological strain upon the human biometeorological system in light of retrieved environmental conditions (Binarti et al. 2020; Jendritzky et al. 2012; Parsons 2003). Such variables enable a more detailed grasp on the interaction with human thermoregulation dynamics (Hensel and Schafer 1984; Katić et al. 2016), and how the human body is generally approached (Giannaros et al. 2018; Holopainen 2012; Höppe 1984; VDI 1998). The six variables obtained directly from the MS were T_a_, relative humidity (RH), vapour pressure (VP), wind speed (V), cloud cover (Oct), and mean radiant temperature (T_mrt_). Retrospectively, the first three variables can be associated to the analytical constituents of the KG, but the addition of V, Oct, and T_mrt_ allows the effects of wind dynamics and radiation fluxes to be considered, which have been fundamental in ‘human-centred approach’ studies. In particular, such ‘bottom-up’ analytical studies have described these variables as the strongest parameters upon urban thermal comfort levels (Algeciras and Matzarakis 2015; Andreou 2013; Charalampopoulos et al. 2016; Hwang et al. 2010; Kántor et al. 2018; Lin 2009; Matzarakis and Amelung 2008; Nouri 2013; Nouri and Costa 2017; Walton et al. 2007).

Although the calculation of the EBM indices does not require the input of both VP and RH, both were included in the study due to their individual implications on local environmental conditions. More specifically, and as discussed by the study undertaken by Nicol (2004), while RH is widely utilised in thermal comfort approaches, its high dependency upon T_a_ enforces its respective ‘relativity’. VP on the other hand is a variable that can better portray evaporative heat loss dynamics, including those associated to human skin surface evaporation patterns as formerly established by Mole (1948) and Brebner et al. (1958). Given their association, such dynamics pertaining to skin temperature (T_Sk_) and perspiration rate (PR) shall be further discussed within the subsequent section.

To avoid imprecision in V speeds that are measured at a different level than that of pedestrian height, in order to ensure that such measurements were pertinent to the gravity centre of the human body, the original values were adapted to a height of 1.1 through application of the formula as defined by Kuttler (2000):

1 $$ {\mathrm{V}}_{1.1}={\mathrm{V}}_h^{\ast }{\left(\frac{1.1}{h}\right)}^{\alpha}\kern1.75em \alpha ={0.12}^{\ast }{z}_0+0.18 $$

where V_h_ is the m/s at a height of *h* (10 m); *α* is an empirical exponent, depending upon urban surface roughness; and *Z*_0_ is the corresponding roughness length. In this study, given the general built-up urban morphological characteristics found in Ankara, *α* was configured at a value of 1.5. As a result, the resulting calibrated wind speed values were henceforth expressed as V_1.1_.

Before moving onto the EBM indices, to obtain an understanding of radiation fluctuations, Oct values were processed in combination with the aforementioned variables within the biometeorological RayMan model to obtain T_mrt_ estimations for each of the measurement hours. Such a parameter determines the radiative exchange between the human body with the encircling environment. Naturally, such a parameter becomes a crucial component in the ‘human-centred approach’, particularly given its intrinsic relationship with built environment elements, including urban morphological dimensions, vegetation, topography, and surface materials (Herrmann and Matzarakis 2012; Nouri et al. 2017; Nouri et al. 2018b; Thorsson et al. 2017; Thorsson et al. 2014).

### Selection of EBM indices

#### PET

The PET index is based upon the Munich energy-balance model for individuals (MEMI) (Höppe 1984; Höppe 1993), and is defined by the T_a_ at which, in a typical indoor setting, the human energy budget is maintained by T_Sk_, core temperature (T_Cr_), and PR are equivalent to those under the assessed conditions. Moreover, and although also based upon a two-node system, it differs from others, such as the Gagge two-node model (Gagge et al. 1986). Generally, the greatest difference between the two can be allocated to the modification in clothing integration. In terms of the implied specific variables, the distinction is attributed to the calculation of the physiological PR as a complementary function of T_Sk_ and T_Cr_, and by the calculation of heat flows from body segments that are covered (or uncovered) by clothing (Höppe 1999). As expected, the type of human activity is also significant, whether it is moving or static (i.e., thus occurring as a result of the energy consumption as a result of metabolic dynamics) which is discussed further for the applied EBMs in the subsequent section after Table 2. Overall, however, and in alignment with the succinct description by Staiger et al. (2019), “[T_a_] is iterated up to a balanced energy budget by adopting the meteorological settings of the reference environment.” (p. 7).

#### mPET

The mPET index, similar to PET, is also upon MEMI principals, but with a few alterations to its intrinsic calculation parameters that have already proved to render slightly different estimations of thermophysiological conditions, particularly in extreme climatic conditions. Comparatively to the original PET index, and according to Chen and Matzarakis (2017), such deviations take place as a result of (i) an integrated multiple-segment thermoregulation model (with a total of 15–25 body model nodes, instead of the original two), and (ii) a clothing model that renders a more accurate analysis of the human bio-heat transfer mechanism. Without discrediting the former EBM index, numerous studies have already documented the augmented capacity of mPET to render more precise thermophysiological estimations based on its enhanced efficiency to identify the human heat transfer dynamics between the inner and outer body (e.g., Charalampopoulos and Nouri 2019; Chen et al. 2020; Lin et al. 2018; Nouri et al. 2017; Nouri et al. 2018b; Şensoy et al. 2020; Wu et al. 2020).

#### UTCI

The third and final EBM index utilised in this study was the UTCI. Although divergent from the MEMI, alike the previous two indices, it is based upon a thermoregulatory model with multiple nodes (187) that describe the human heat transfer and thermoregulatory system, known as the UTCI-Fiala model (Fiala et al. 2012). Intrinsic to this approach is the termed ‘local’ (or adaptive) clothing model that considers implications of the different clothing arrangements/combinations based upon the observed clothing patterns worn by Europeans (Havenith et al. 2012). Again, similar to the PET and the mPET indices, the input requirements are also based upon the aforementioned singular variables as confirmed in the algorithms as described by Bröde et al. (2012).

#### Identification and adaptation of PS grades

As stated, all of the three EBM indices are extensively used within thermophysiological studies. In this study, their respective selection was also attributed to their (i) feasibility in being calibrated on easily obtainable singular climatic variables, and (ii) their base measuring unit being (°C), thus simplifying their interpretation for non-climatic experts such as architects, urban planners, and designers to apply such information when approaching urban environmental conditions. Furthermore, based upon a ‘human-centred approach’, each of the EBM indices can be cross-examined against respective quantitative PS thresholds as illustrated in Table 2. Within the table, while the two types of EBM indices cannot be directly compared due to their inbuilt calculation methods, it demonstrates the respective relationships between the three indices and their intrinsic PS thresholds.

**Table 2:** Relationship between PET, mPET, and UTCI against respective PS stress levels, each based on the MEMI and Fiala model, respectively (source: adapted from Matzarakis et al. (1999) and Bröde et al. (2012))

(°C)	PS level	Stress level abr.		Existing/added
MEMI-based EBM				
PET/mPET*				
< − 20	Beyond extreme cold stress 3	Cold stress	(CS7)	Added
− 20~− 10	Beyond extreme cold stress 2		(CS6)	Added
− 10~0	Beyond extreme cold stress 1		(CS5)	Added
0~4	Extreme cold stress		(CS4)	Existing
4~8	Strong cold stress		(CS3)	Existing
8~13	Moderate cold stress		(CS2)	Existing
13~18	Slight cold stress		(CS1)	Existing
18~23	No thermal stress	(−)	(NS)	Existing
23~29	Slight heat stress	Heat stress	(HS1)	Existing
29~35	Moderate heat stress		(HS2)	Existing
35~41	Strong heat stress		(HS3)	Existing
41~46	Extreme heat stress		(HS4)	Added
> 46	Beyond extreme heat stress		(HS5)	Added
Fiala model-based EBM				
UTCI**				
< − 40	Extreme cold stress	Cold stress	(CS5)	Existing
− 40~− 27	Very strong cold stress		(CS4)	Existing
− 27~− 13	Strong cold stress		(CS3)	Existing
− 13~0	Moderate cold stress		(CS2)	Existing
0~9	Slight cold stress		(CS1)	Existing
9~26	No thermal stress	(−)	(NS)	Existing
26~32	Moderate heat stress	Heat stress	(HS1)	Existing
32~38	Strong heat stress		(HS2)	Existing
38~46	Very strong heat stress		(HS3)	Existing
> 46	Extreme heat stress		(HS4)	Existing

*Ranges of PS for PET and mPET calculation based upon an internal heat production of 80 W, and a heat transfer resistance of the clothing set to a value of 0.9 clo according to Matzarakis and Mayer (1997)

**Ranges of PS for UTCI calculation based upon an internal heat production of 135 W, with an adaptive clothing model as stipulated by Havenith et al. (2012)

In the case of PET and mPET, within this study, and in alignment with various studies that have discussed the relationship/calibration of thermophysiological indices against their originally designated PS and/or thermal perception grades (e.g., Hwang and Lin 2007; Lin 2009; Lin and Matzarakis 2008; Nouri et al. 2017; Potchter et al. 2018), the original grades as proposed by Matzarakis et al. (1999) were synoptically extended. While undoubtedly requiring further study to mature the relationship between the thermophysiological indices (particularly in the case of mPET on account of its deviations from the original MEMI model, and later arrival to the scientific community in 2017), the extension permitted the authors to better plot PET/mPET values that either went considerably below 4 °C or above 41 °C. Nevertheless, the extensions on both extremes were methodically approached differently.

With regard to heat stimulus, and grounded upon the exploratory ‘What if?’ approach for extreme environmental scenarios as conducted by Nouri et al. (2018c) and Nouri et al. (2018a), based upon an increment of roughly 5 °C per physiological threshold within the existing grade system, two new grades were adapted beyond the original ‘extreme heat stress’ classification (> 41 °C). Attributed to them being the fourth and fifth level of heat stress (HS) after the ‘no thermal stress’ classification, such new grades were designated as HS4 and HS5 according to the stress level abbreviation system as depicted in Table 2.

The objective of the abbreviation system was intended to (i) reduce/condense the amount of terminology utilised when referring to each of PS levels within the results/discussion section of the paper, and (ii) to highlight that given the extended expansion from the original levels as originally described by Matzarakis et al. (1999), further study is required to indicate the correct terminology to describe such stimulus upon the human biometeorological system.

Concerning cold stress, the issue of terminology became particularly palpable in light of the three added cold stress (CS) levels (i.e., CS5, CS6, and CS7) ‘beyond’ the original ‘extreme cold stress’ threshold. These added PS grades were established upon increments of 10 °C (when below 0 °C) as also applied in the study undertaken by Matzarakis (2014a) in the German municipality of Sankt Peter-Ording to better plot PET values below 4 °C. Given augmented vulnerability to CS, as also suggested by the KG classifications indicated in Table 1 for Ankara, such an increment was extended an additional level in order to determine PET/mPET values that went below − 20 °C. Naturally, the terminology of ‘beyond extreme cold stress 1–3’ requires further study to better describe, in terminological terms, the physiological stimulus that the environmental conditions can have upon the human biometeorological system. Nevertheless, and in light of the this study’s objective to identify/plot such frequencies, and breadth, of such extreme CS/HS events, the synoptic expansion of the PS grades enabled a more encompassing delimitation of Ankara’s thermophysiological conditions during both the summer and winter months.

Lastly, and as noted within Table 2, both the MEMI and Fiala model are, in general terms, based upon different clothing resistance (clo) and internal heat production values. As an example, the basic configuration of mPET/PET calculation is established upon a standing human being, while the UTCI is based upon a person walking (hence, the augmented heat production, which in the case of PET/mPET is resultant of the human metabolism system, rather than caloric expenditure as a result of physical activity in the case of UTCI). Consequently, and in the interest of consistency, the basic configuration of UTCI was constituted within the RayMan model to be the same as those for PET and mPET.

### Temporal period and meteorological station selection

So far, the methodology of calculating EBM indices from singular variables retrieved from MSs has been widely utilised in thermal comfort and environmental studies. Undertaken in a wide array of KG classification typologies, and for different analytical temporal periods, a selection of such studies were summarised in Table 3.

**Table 3:** Summary of 20 selected studies using MS station data to undertake human EBM indices

#	Country	City	KG class.	Principal EBM index	Survey period	Total years	Analysis season	Source
1	Taiwan	Sun Moon Lake	‘*Cwa*’	PET	1996–2005	10	Annual	Lin and Matzarakis (2008)
2	Taiwan	Taichung City	‘*Cwa*’	PET	2007–2008	1	1 hot and 1 cold season	Lin (2009)
3	Taiwan	Keelung, Taichung, Tainan	‘*Cfa*’, ‘Cwa’, ‘*Cfa/Aw*’	PET	2011–2014	4	Hot/cold Seasons	Lin et al. (2015)
4	Taiwan	Huwei Township	‘*Cwa*’	PET	2000–2009	10	Annual	Hwang et al. (2010)
5	China	NA	11 class.	PET	2000–2012	11	Annual	Yang and Matzarakis (2016)
6	Germany	Freiburg	‘*Cfb*’	PET	1999–2009	10	Annual	Herrmann and Matzarakis (2012)
7	Germany	Freiburg	‘*Cfb*’	PET, UTCI, PT	1999–2010	11	Annual	Fröhlich et al. (2019)
8	Turkey	Ankara	–	PET	2001–2010	10	Annual	Türkoğlu et al. (2012)
9	Turkey	Ankara	–	PET	1975–2013	38	Annual	Çalışkan and Türkoğlu (2014)
10	Turkey	Antalya	‘*Csa*’, ‘*Csb*’, ‘*Dsb*’	PET, mPET	1960–2017 (CNTRL)	58	Annual	Şensoy et al. (2020)
11	Turkey	Istanbul	‘*Csa*’	PET	2000–2006	7	Annual	Matzarakis and Karagülle (2007)
12	Turkey	Bursa	‘*Csa*’	PET	1975–2006	31	Annual	Çalışkan et al. (2012)
13	Turkey	Erzurum Plain	‘*Dfb*’	PET	2009–2010	2	Annual	Yilmaz et al. (2013)
14	Greece	Athens	‘*Csa*’	PET, mPET, UTCI, PT	2002–2008	7	Annual	Charalampopoulos (2019)
15	Greece	Athens	‘*Csa*’	PET, mPET, UTCI, PT	2002–2016	15	Annual	Charalampopoulos and Nouri (2019)
16	Greece	Evros	‘*Csa*’	PET	1961–1990	30	Annual	Matzarakis (2007)
17	Portugal	Lisbon	‘*Csa*’	PET, mPET	2012–2016	5	Hot season	Nouri et al. (2017)
18	Portugal	Lisbon	‘*Csa*’	PET	2001–2002	1	1 hot/cold season	Alcoforado and Andrade (2007)
19	Canada	Quebec City	‘*Dfb*’	UTCI, PET	2013–2014	1	Annual	Provençal et al. (2016)
20	Iran	Kerman	‘*BWk*’	PET, UTCI, SET*	2016	1	Annual	Zare et al. (2018)

All of the disclosed twenty studies focused upon the application of EBM indices, including those not included in this study, namely the perceived temperature (PT) (Staiger et al. 2012) and standard effective temperature (SET*) (de-Dear and Brager 1998; Gagge et al. 1986; Gonzalez et al. 1974). In the case of the latter, it can also be noted that SET* is based upon the previously mentioned Gagge’s two-node model and applied PR. Although based upon similar methodologies and/or variables, the identified studies were focused on numerous intentions, topically ranging from ascertaining the (i) implications upon tourism and communication of bioclimatic information (table study nos. 1, 10–12, 16–17); (ii) influences of human thermal perception on index application and classification (table study nos. 2–3); (iii) relationship with urban structures/topography and respective modification of index values (table study nos. 4, 6, 7–9, 13, 17–18); (iv) methodical application of modelling packages to determine index values (table study no. 7); (v) suitability and comparison between evaluated indices (table study nos. 14–15, 19–20); and (vi) potential symbiotic relationships between KG classifications and respective indices (table study no. 5).

**Fig. 1:**
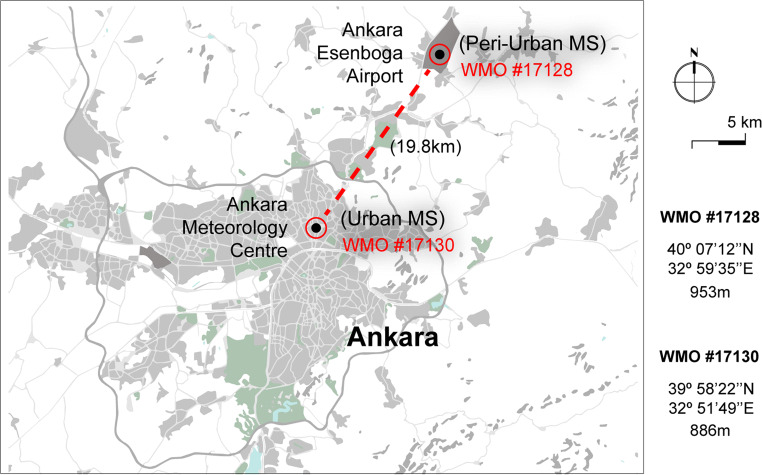
Location of the two meteorological stations located in the city’s urban meteorology centre (#17130) and in the peri-urban Ankara Esenboga Airport (#17128)

Given the objectives of this specific study, a temporal analytical period of 10 years was selected, which enabled a suitable timeframe to account for yearly oscillations and identification of both cold and heat extremes in the past decade for Ankara. Moreover, the study retrieved data from two stations in order to also examine/identify thermophysiological variability within the urban city centre through the WMO’s MS N°17130 (at a latitude of 39° 58′ 22″ N, longitude of 32° 51′ 40″ E, and an altitude of 886 m ASL), and the peri-urban setting as portrayed by Ankara’s Esenboga Airport MS N°17128 (at a latitude of 40° 07′ 12″ N, longitude of 32° 59′ 35″ E, and an altitude of 953 m ASL) (Fig. 1). For the case of Ankara, and distanced approximately 20 km away from one another, both the urban MS and peri-urban MS were those which moreover contained the most amount of meteorological data, fewer calibration periods, and lesser intervals between measurement periods over the past decade.

### Representation of results

With the aim of certifying the easy interpretation of the results obtained by this study, two principal methods were utilised to present the obtained thermophysiological outcomes. Subsequently to the meteorological tables which depict the minimum, mean, and maximum measurements for both stations for each season during the past decade, the Climate-Tourism/Transfer-Information-Scheme (CTIS) (Matzarakis 2014b) and frequency distribution diagrams (FDDs) were utilised. Correspondingly, such communication methodologies were commonly applied in international studies as exemplified in Table 3. In the case of the CTIS, based on the calibration of the chosen thresholds and temporal period, the software enabled the graphical representation of variable fluctuations, thus aiding the interpretation of the bioclimatic outcomes. To comply with the grades presented in Table 2, the colour coding was based upon the upper limits of each respective PS level for the three EBM indices. Pertaining to the FDDs, they enabled the communication of the frequency percentages of respective PS occurrence at a specific time of day. In this study, the FDD hours were set at 03:00 to represent nocturnal variations of PS, and 15:00 to portray the same variations during the day for both stations for the assessed indices. Correspondingly, such hours also frequently corresponded with the coldest hour and hottest hour for each day, further enhancing their applicability to identify cold/heat stress frequencies.

## Results

### Singular variables

Within this section, the presented meteorological tables illustrate the measurements obtained for both the peri-urban and urban MSs. Although the variables were collected at a 3-h interval, the tables in this section were condensed to focus instead on four critical hours abridging two nocturnal and two diurnal measurements. Based upon an average of all years between 2010 and 2019, the $\overline{\operatorname{Min}.}$, $\overline{\mathrm{Mean}.}$, and $\overline{\operatorname{Max}.}$ for each season was presented for the aforementioned 6-h interval period pertaining to each of the singular variables. For both MSs, in the case of T_a_, and as demonstrated in Table 4 (A), it was possible to identify four seasons, and the considerable range of temperatures between the DJF and JJA periods. The $\overline{\operatorname{Min}.}$ recorded T_a_ for the past decade was of − 22.5 °C recorded by the peri-urban MS for the DJF season. On the other hand, the $\overline{\operatorname{Max}.}$ T_a_ was of 39.9 °C recorded by the urban MS during the JJA months. It was also noted that $\overline{\operatorname{Max}.}$ values were also considerably high for the SON season which is due to September still recording high temperatures with a considerable subsequent drop in October. Between the two stations, it was clear that the peri-urban MS in the Esenboga Airport presented lower T_a_ values in comparison to those registered in the city centre. Such variations can partially be attributable to urban heat island (UHI) effect that rendered differences in $\overline{\mathrm{Mean}.}$ values by up to 3.4 K recorded during the MAM months.

**Table 4:** Average seasonal variations of (A) T_a_ (°C) | (B) V_1.1_ (m/s) | (C) RH (%) | (D) VP (hPA) | (E) T_mrt_ (°C) during the last decade with a 6-h measurement interval for both the peri-urban and urban meteorological stations

		Winter (DJF)				Spring (MAM)				Summer (JJA)				Autumn (SON)			
		03:00	09:00	15:00	21:00	03:00	09:00	15:00	21:00	03:00	09:00	15:00	21:00	03:00	09:00	15:00	21:00
Peri-urb.	Ta $\overline{\operatorname{Min}.}$	− 22.0	− 22.5	− 9.4	− 15.2	− 10.6	− 10.2	− 3.4	− 5.7	6.2	11.4	10.9	10.8	− 9.7	− 10.0	1.9	− 4.7
	Ta $\overline{\mathrm{Mean}}$	− 1.0	− 1.5	5.0	1.3	5.2	7.7	14.8	10.0	16.3	20.6	27.6	22.3	8.5	9.9	19.5	13.1
	Ta $\overline{\operatorname{Max}.}$	11.4	14.0	20.1	12.5	18.5	22.8	31.4	23.9	28.1	30.1	38.8	33.1	28.1	30.1	37.9	32.9
Urban	Ta $\overline{\operatorname{Min}.}$	− 14.5	− 15.7	− 7.1	− 10.0	− 5.9	− 5.4	− 2.4	− 3.0	9.8	11.7	13.3	12.3	− 4.4	− 7.1	3.2	− 0.2
	Ta $\overline{\mathrm{Mean}}$	1.3	0.8	6.3	3.4	8.6	10.8	16.8	33.7	18.8	22.4	29.0	24.5	10.7	11.9	19.7	14.5
	Ta $\overline{\operatorname{Max}.}$	13.8	15.9	20.8	14.7	21.9	25.4	33.7	28.7	29.2	31.0	39.9	37.3	24.2	28.0	37.6	29.1
Peri-urb.	V_1.1_ $\overline{\operatorname{Min}.}$	0.0	0.0	0.0	0.0	0.0	0.0	0.3	0.0	0.0	0.0	0.0	0.0	0.0	0.0	0.0	0.0
	V_1.1_ $\overline{\mathrm{Mean}}$	1.2	1.2	2.2	1.5	1.2	1.7	2.9	1.8	1.6	1.7	2.7	2.6	1.2	1.2	2.4	1.7
	V_1.1_ $\overline{\operatorname{Max}.}$	7.1	6.7	8.8	7.7	8.4	7.4	10.4	9.4	4.7	6.1	9.4	7.7	6.1	7.1	7.4	6.7
Urban	V_1.1_ $\overline{\operatorname{Min}.}$	0.0	0.0	0.0	0.0	0.0	0.0	0.3	0.0	0.0	0.3	0.0	0.0	0.0	0.0	0.0	0.0
	V_1.1_ $\overline{\mathrm{Mean}}$	1.1	1.1	1.4	1.1	1.1	1.2	1.8	1.3	1.5	1.5	1.8	1.9	1.2	1.3	1.3	1.1
	V_1.1_ $\overline{\operatorname{Max}.}$	5.1	5.1	5.7	5.4	5.4	6.7	7.7	5.4	4.4	5.1	6.4	5.4	5.4	6.4	5.4	5.1
Peri-urb.	RH $\overline{\operatorname{Min}.}$	45	40	14	30	28	17	9	17	24	17	7	11	24	16	7	12
	RH $\overline{\mathrm{Mean}}$	88	89	64	80	79	70	43	61	69	54	29	46	73	69	37	57
	RH $\overline{\operatorname{Max}.}$	100	100	100	100	100	100	100	97	96	99	88	96	100	100	95	100
Urban	RH $\overline{\operatorname{Min}.}$	37	33	14	28	24	2	2	2	20	22	3	6	23	19	5	10
	RH $\overline{\mathrm{Mean}}$	83	85	63	75	71	65	41	53	62	51	28	41	68	65	38	54
	RH $\overline{\operatorname{Max}.}$	100	100	100	100	100	100	100	99	100	99	100	99	99	100	99	99
Peri-urb.	VP $\overline{\operatorname{Min}.}$	0.9	0.8	1.4	1.7	2.6	2.6	2.0	2.3	4.1	5.0	2.9	3.9	1.4	1.5	1.9	1.6
	VP $\overline{\mathrm{Mean}}$	5.2	5.1	5.6	5.5	7.5	7.9	7.1	7.8	12.6	12.8	10.1	11.8	7.9	8.2	7.5	8.0
	VP $\overline{\operatorname{Max}.}$	9.7	10.5	12.7	10.7	14.5	14.6	16.7	17.3	18.8	20.1	17.6	19.3	16.8	18.2	16.7	16.8
Urban	VP $\overline{\operatorname{Min}.}$	1.6	1.4	1.2	1.6	2.4	0.2	0.2	0.2	4.3	6.9	1.6	2.6	2.0	1.9	1.8	2.5
	VP $\overline{\mathrm{Mean}}$	5.8	5.7	6.0	6.0	8.1	.8.4	7.4	7.8	13.3	13.6	10.6	11.7	8.8	9.0	8.0	8.6
	VP $\overline{\operatorname{Max}.}$	11.1	11.3	13.1	11.1	15.1	16.4	17.3	17.7	22.1	22.5	19.1	21.8	17.3	16.9	20.3	18.4
Peri-urb.	Tmrt $\overline{\operatorname{Min}.}$	− 37.6	− 32.6	− 15.9	− 29.4	− 24.0	− 16.0	− 6.0	− 18.5	− 3.8	16.1	16.2	4.1	− 23.9	− 21.2	3.3	− 18.0
	Tmrt $\overline{\mathrm{Mean}}$	− 9.4	1.8	11.3	− 6.9	− 2.9	30.5	33.3	2.7	8.0	45.7	47.3	14.3	− 1.4	22.8	28.6	3.4
	Tmrt $\overline{\operatorname{Max}.}$	5.8	22.7	32.2	6.3	11.6	47.6	49.9	16.3	21.8	54.3	59.0	25.0	16.3	49.2	50.1	20.3
Urban	Tmrt $\overline{\operatorname{Min}.}$	− 28.4	− 19.3	− 10.6	− 24.0	− 18.1	− 7.8	− 4.8	− 14.0	0.2	16.5	35.1	5.2	− 17.8	− 16.3	2.5	− 12.8
	Tmrt $\overline{\mathrm{Mean}}$	− 6.9	6.0	13.2	− 4.8	0.0	31.5	34.7	4.7	10.6	47.1	49.4	16.2	1.8	27.8	30.2	5.7
	Tmrt $\overline{\operatorname{Max}.}$	7.6	25.2	33.7	10.4	18.0	49.8	52.8	18.8	20.9	56.9	59.3	28.4	16.1	48.5	52.5	21.0
		03:00	09:00	15:00	21:00	03:00	09:00	15:00	21:00	03:00	09:00	15:00	21:00	03:00	09:00	15:00	21:00
		Winter (DJF)				Spring (MAM)				Summer (JJA)				Autumn (SON)			

By a substantial amount, it was possible to verify that obtained $\overline{\operatorname{Max}.}$ (and even $\overline{\mathrm{Mean}.}$) T_a_ surpassed the values associated to a ‘*Dsb*’ KG classification as disclosed in Table 1, and resemble more that of a hot summer climate (i.e., that of a ‘*Dsa*’ classification) where temperatures surpass the labelled T_hot_ of 22 °C. This being said, and noting that the snow/cold climate ‘*Dsa*’ T_cold_ threshold is based upon its coldest month being ≤ 0 °C, such a classification might also not be the most suitable. While the month of January revealed a $\overline{\operatorname{Min}.}$ T_a_ of − 19.5 °C, the $\overline{\mathrm{Mean}.}$ T_a_ at 15:00 registered by the urban MS was of 4.1 °C, and the $\overline{\operatorname{Max}.}$ was 15.3 °C for the same hour. On the one hand, while these results would be more suitable to the ‘*Csa*’ T_cold_ thresholds of < 18 °C, such a classification would not be compatible with the obtained $\overline{\operatorname{Min}.}$ values for T_a_. Finally, the ‘*BSk*’ that is less specific about its seasonal characteristics is based upon mean annual temperatures being < 18 °C, in the case of the peri-urban MS, the $\overline{\mathrm{Mean}.}$ was of 11.2 °C, and the urban MS presented a $\overline{\mathrm{Mean}.}$ of 13.3 °C. Nevertheless, to confirm such a classification, P_R_ measurements also need to be considered in conjunction with the measured T_a_ values in a future study.

As shown in Table 4 (B), V_1.1_ did not show the same type of seasonal variation as demonstrated by T_a_. However, the peri-urban MS generally revealed slightly higher values in comparison to those registered in the city centre. Again, the identified variations can be linked to the differences between the peri-urban and urban morphological characteristics which influence resulting wind permeation patterns at each MS. In broad terms, two general similarities were identified for both stations: (1) $\overline{\mathrm{Mean}.}$ values were generally low for all seasons, and (2) 15:00 was the hour which almost always revealed the highest V_1.1_ values.

With regard to RH (Table 4 (C)) and VP (Table 4 (D)), the meteorological tables revealed different environmental information from one another. On the one hand, RH could be better interpreted through its $\overline{\mathrm{Mean}.}$ values due maximum percentages being a frequent occurrence during days with high P_R_ and/or heavy fog. More specifically, it is well known that the months of December, January, and May are the months with the heaviest rainfall patterns, yet (particularly in the case of May) such patterns are amalgamated with the other seasonal months. Nevertheless, the DJF season presented the highest $\overline{\mathrm{Mean}.}$ percentages at both stations, which naturally accounts for higher atmospheric water content associated to rain/snow fall and lower T_a_ values. Reversely, the JJA and SON seasons presented the lowest percentages, particularly in the afternoon and late evening hours. Such patterns were shared between the two MSs.

In comparison to RH, VP presented much clearer differentiations between the different seasons, and between the two MSs. When considering the DJF season, hPA values were considerably lower due to the lower atmospheric condensation rates in comparison to the JJA months. During the summer, a $\overline{\mathrm{Mean}.}$ range between 10.1 and 13.6 hPA was identified, which in comparison, were similar to the $\overline{\operatorname{Max}.}$ values retrieved for the DJF months. When considering such implications upon the human biometeorological systems, the higher hPA values retrieved for the JJA period (and further heightened at the urban MS) indicated a greater dehydration risk as a result of the higher saturation deficit during the hotter months of the year. Correspondingly, the increased hPA values presented by the urban MS can be further interconnected with the UHI crescendo as specified by Holmer and Eliasson (1999). In terms of human health risk factors, in addition to the physiological strain of T_a_ upon the biometeorological system, such atmospheric conditions further augment the expected susceptibility to heat and/or sunstroke occurrences.

By processing Oct values with the aforementioned variables within the RayMan model, it was feasible to calculate average seasonal T_mrt_ outcomes as presented in Table 4 (E). Similar to T_a_, V_1.1_, and VP, it was also possible to ascertain clear distinctions between the two MSs, where the urban MS presented generally greater T_mrt_ values. Additionally, and alike T_a_ and V_1.1_, 15:00 was the hour with the highest T_mrt_ measurements. As expected, the circadian fluctuations in solar radiation resulted in significant variations between the nocturnal and diurnal measurements (e.g., between 03:00 and 15:00). Such a result is directly attributable to the increased exposure to radiant (both short-wave and long-wave) heat exchange between the human biometeorological system with that of the surrounding environment.

Just as importantly, and heightening the crucial aspect of the ‘human-centred approach’ applied in the study, the clear resulting discrepancy between T_a_ and T_mrt_ values highlighted the importance of radiation when approaching thermal comfort thresholds. Although not an index, the inclusion of such a non-temperature variable enabled the comprehension of how radiation flux played a paramount role in approaching both HS and CS. In the case of DJF, it is possible to verify that T_mrt_ went well below those of T_a_ in the case of $\overline{\operatorname{Min}.}$ values. Nevertheless, during the day, even with the winter sun (consisting of far lower incident solar radiation levels), both $\overline{\mathrm{Mean}.}$ and $\overline{\operatorname{Max}.}$ T_mrt_ values were considerably higher as a result the exposure to diurnal radiant heat. Opposingly, during the summer season, and during the same diurnal hours, both $\overline{\mathrm{Mean}.}$ and $\overline{\operatorname{Max}.}$ T_mrt_ values revealed vast variations from the T_a_ measurements. This being said, during the nocturnal summer period, T_a_ always surpassed T_mrt_ as a result of the aforementioned lack of radiant heat exchange with the human body.

### EBMs

#### CTIS analysis

**Fig. 2:**
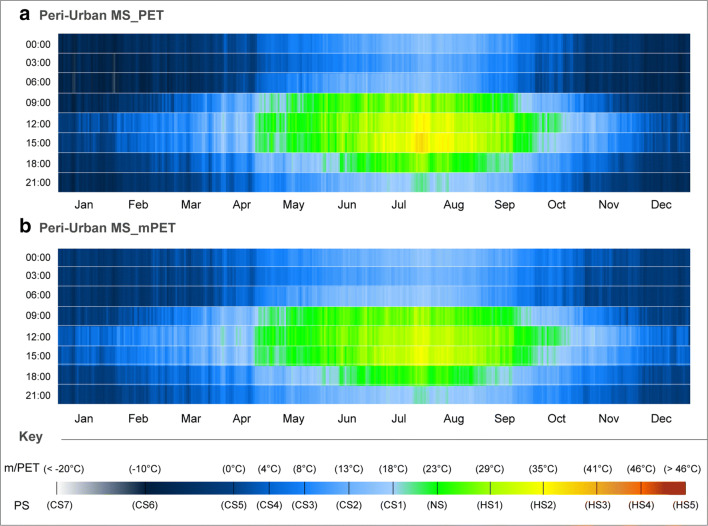
CTIS diagram of average daily PS grades for PET and mPET between 00:00 and 21:00 with a 3-h interval relative to the peri-urban MS for the past decade in Ankara. **a** Peri-urban MS_PET. **b** Peri-urban MS_mPET

Returning to the 3-h measurement interval for the EBM results, Fig. 2 demonstrated the daily bioclimatic variation of PS between for both the PET and mPET indices for the peri-urban MS over the past decade. From the end of April onwards until the end of October, the diurnal hours revealed PS levels fluctuating between NS and HS2. Nevertheless, unlike mPET, PET reached the HS3 as a result of values surpassing the 41 °C threshold during late July and early August. Moreover, the PET index presented a higher frequency of HS2 for the same period, thus suggesting generally higher susceptibility levels to HS during the summer period.

Amid all of the hours, and as linked to the greater exposure to radiant heat exchange, 12:00 and 15:00 were the hours with the highest levels of HS all year round. Naturally, as the days became longer, the hours of 09:00 and 18:00 followed a similar PS distribution between late spring and early autumn. Nevertheless, the 18:00 measurements only indicated NS predominantly between the summer months, with a minor extension to the 21:00 measurements, likely due to enduring high T_a_ values after sunset. It was also noted that even during the peak of the summer, other nocturnal measurement hours always revealed some level of CS, particularly at 03:00 that still remained between CS2 and CS3.

During the DJF period, PET levels revealed significantly different PS conditions. As exemplified in January, it was possible to identify that over the past 10 years, even during the day, PS levels would remain between CS3 and CS5. Moreover, January also revealed the occurrence of extreme cold events between the hours of 03:00 and 06:00 where PS reached CS7. As the CTIS image is based upon the averages of each yearly dataset between 2010 and 2019, the manifestation of such extreme cold thermophysiological conditions in Fig. 2 a indicated that specific individual years would expose significantly longer durations/intensities of CS7.

Opposingly, mPET results revealed two principal differences from the PET index, namely the more attenuated levels of (i) HS during the summer, which did not exceed HS2; and (2) CS during the winter which very rarely went below CS5. Such results highlight the tendency of the updated EBM index to present lower PS estimations during periods of more elevated human thermophysiological stress. This acknowledged comportment can likewise explain the higher frequency of both CS1 and HS1 in Fig. 2 b.

**Fig. 3:**
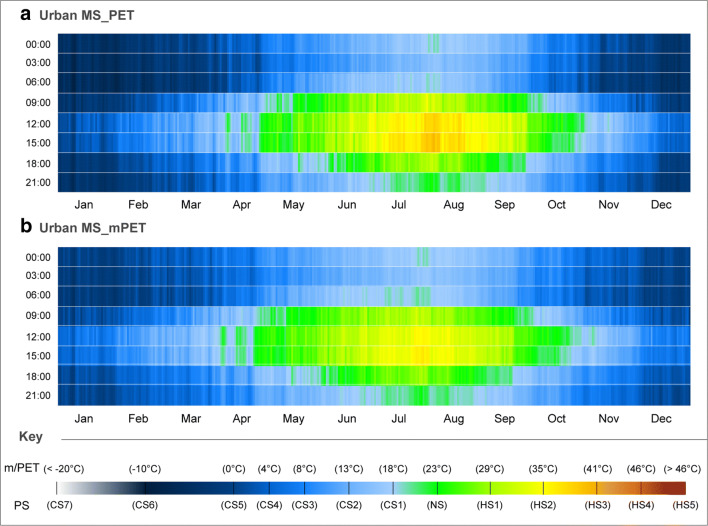
CTIS diagram of average daily PS grades for PET and mPET between 00:00 and 21:00 with a 3-h interval relative to the urban MS for the past decade in Ankara. **a** Urban MS_PET. **b** Urban MS_mPET

In comparison to the peri-urban MS, Fig. 3 revealed that the urban MS station generally presented higher extents of HS during all seasons. As presented in Fig. 3 a, the average occurrence of HS3 increased in frequency between the months of July and August, hence suggesting the vulnerability to the occurrence of extreme heat events such as heat waves. Moreover, and unlike in the previous peri-urban station, the diurnal periodicity of average HS3 in the city centre was generally longer as the 12:00 also revealed such PS levels. Moreover, the frequency of days vulnerable to HS3 also verified to be an issue of concern as it remained nearly constant between mid-July and mid-August. Similar to the aforementioned CS extremes during the winter period in Fig. 2 a, individual year datasets were certainly going to reveal higher exposure levels to HS.

In addition to the increased vulnerability to HS3 during July and August, it was also noted that the average frequency of HS2 remained practically constant at 12:00 and 15:00 from mid-June until the end of September. Considering that such a PS level is concomitant to a PET of 35 °C, this correlates to a considerably prolonged period with elevated vulnerability to HS during the early afternoon. The nocturnal hours (with the exception for 03:00) also revealed periods of NS during the peak of the summer period.

Between the summer and winter period, it was possible to identify a slightly more gradual transition in PS levels. Unlike with the peri-urban MS, CS1 and CS2 were observed more frequently during the winter, especially during the middle of the day. In addition, while periods of CS6 were observed for the urban MS, their frequency was lower, hence the CTIS revealing a slightly lighter blue hue, without any CS7 aggravations.

Once again, and as illustrated in Fig. 3 b, it was possible to verify that mPET further attenuated both CS and HS for the winter and summer periods, respectively. Comparatively, mPET presented somewhat similar results to those obtained for the peri-urban MS with the following divergences: (i) short period between July and August where PS levels indicated slight vulnerability to HS3 during the early afternoon; (ii) a considerably longer and more consistent exposure to HS2 between June and September—thus in part resembling the aforementioned prolonged exposure that was also identified for PET. Finally, and out of the four CTIS diagrams, mPET in the urban MS presented the most attenuated PS levels during the winter period, with fewer descents from CS5.

**Fig. 4:**
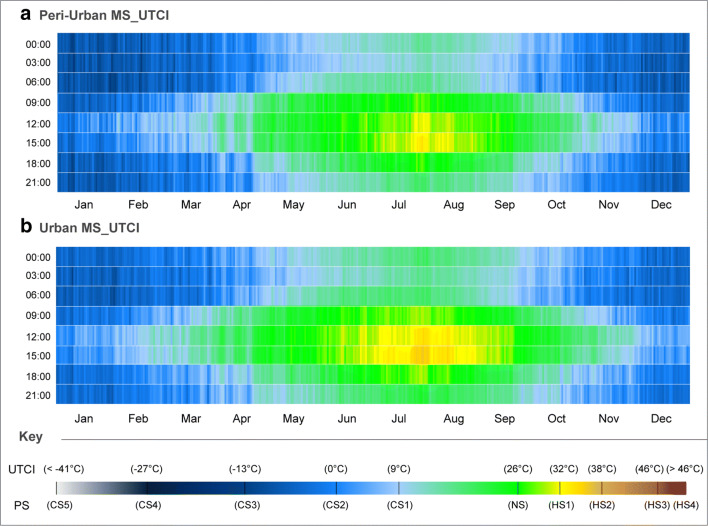
CTIS diagram of average daily PS grades for UCTI between 00:00 and 21:00 with a 3-h interval relative to peri-urban and urban MSs for the past decade in Ankara. **a** Peri-urban MS_UTCI. **b** Urban MS_UTCI

Generally, the obtained UTCI measurements presented different results from the both PET and mPET in terms of annual PS distribution (Fig. 4 a, b). Beyond the divergences between their algorithmic calculation procedures, the differences in PS thresholds for UTCI induced considerable differences in the acknowledged vulnerabilities both to HS and CS. Due to the larger breadth between NS and CS1 (with a span of 15 °C), a much larger portion of PS vulnerability sat between these classifications. In addition, higher PS levels for both CS and HS were also less frequent at both stations. It was nevertheless noted that while HS limits were based upon a common value of > 46 °C (even if attributed to a different HS class), lowest CS boundaries were based upon much lower value of < − 41 °C (instead of < − 20 °C for the MEMI-based indices).

This being said, it was noted that the peri-urban MS did present slightly higher CS during the winter months, and the urban MS revealed higher HS between July and August. In reflection with the two previous EBM index results, UTCI revealed that (i) the urban MS continued to reveal higher HS during both the winter and summer seasons, and (ii) although such comparisons are relative, and must be approached with caution, UTCI tended to present more comparable results to PET, particularly during the summer months. Finally, and in comparison to both the PET and mPET outcomes, the CTIS analysis of UTCI proved to be more complicated, which in turn rendered the diagrams to appear more ‘choppy’. Such a complication originated when the introduced singular V_1.1_ values were ≤ 0.3 m/s, as this impeded the RayMan model to calculate UTCI during such environmental conditions. Resultantly, when V_1.1_ were close to $\overline{\operatorname{Min}.}$ values, it was not possible to obtain UTCI results, hence leading to (i) incomplete yearly averages in the CTIS analysis, and (ii) the impossibility to approach specific (and not average) time frames due to the elevated frequency of hourly measurements where V_1.1_ values were ≤ 0.3 m/s.

**Fig. 5:**
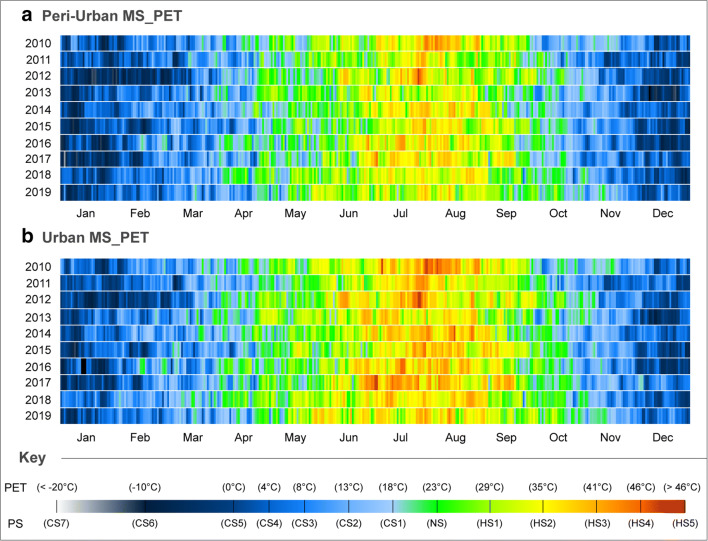
CTIS diagram of daily PS grades for PET at 15:00 for each year in the peri-urban/urban MSs. **a** Peri-urban MS_PET. **b** Urban MS_PET

Within the previous CTIS diagrams, it was possible to ascertain the resulting average PS vulnerability with an interval period of 3 h during the last decade for each of the applied EBM indices. Nevertheless, as identified in the previous diagrams, this had the downside of obscuring extreme HS and CS levels during the individual years. Accordingly, and utilising the PET index, a daily CTIS analysis conducted at 15:00 for each of the year between 2010 and 2019 was undertaken. As demonstrated in Fig. 5, such a diagram enabled a better understanding of resulting PS vulnerability for each of the analysed years. Immediately, it was possible to confirm the greater HS vulnerability during the summer period with PS levels increasing all the way up to HS4 and HS5 for considerable temporal periods as exemplified in 2010, 2012, and 2017 by the urban MS. In thermophysiological terms, such extreme heat events are crucial in the identification to biometeorological vulnerability, including heat-related mortality and morbidity in the city centre of Ankara. In accumulation, it is imperative to value the mixture between the occurrences of very hot days amid the ongoing heatwaves, which further exacerbate the already high ongoing circadian cumulative thermal stress levels, thus raising such urban health and safety concerns even further.

In comparison, although the peri-urban MS still presented high HS conditions as exemplified by the 2010 and 2012 datasets, PS levels were generally more attenuated during the summer period (including with a higher frequency of NS grades). Such conditions sharply contrasted the almost continuous vulnerability to at least HS1 and HS2 registered by the urban MS. Nevertheless, although at 15:00, traces of extreme CS were acknowledged during the winter (as shown mid-January in 2012, early January in 2017, and late December in 2013). These results indicated that even during the hottest measurement hour, it was still possible to identify extreme CS levels, hence serving as a tell-tale sign for the greater vulnerability during the nocturnal period for such years in Ankara.

**Fig. 6:**
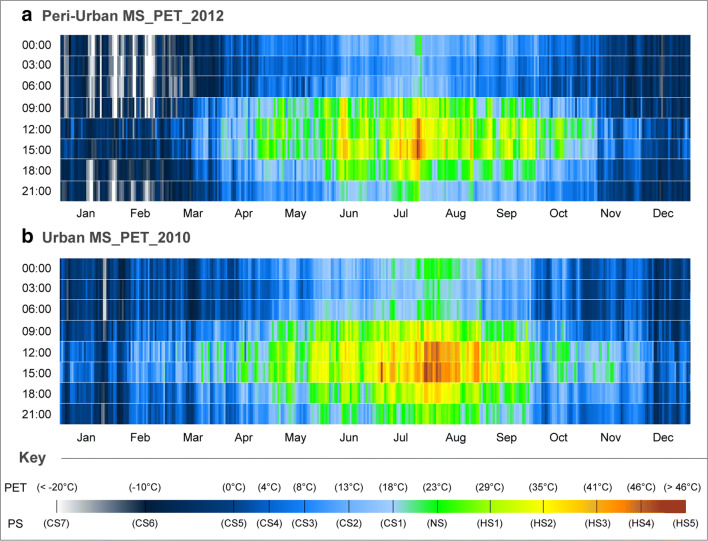
CTIS diagram of daily PS grades for PET at a 3-h interval for individual years of **a** 2012 for the Peri-urban MS and **b** 2010 for the Urban MS

In collaboration with the recognized HS implications upon human biometeorological factors, vigilance must also be considered for the identified thermophysiological risk factors during these periods with extreme CS levels. These periods also present considerable adverse CS impacts upon human wellbeing and safety during the winter, especially as such risk factors are moreover not only limited to the nocturnal period. Albeit, and to further analyse such extreme PS risk factors, the CTIS analysis results for the individual 2010 and 2012 datasets were presented in Fig. 6.

As confirmed in Fig. 6a, the CTIS results revealed considerable nocturnal exposure to elevated CS levels frequently below < − 20 °C during the winter period in 2012 for the peri-urban MS. More specifically, in the first week of February, the measurement hours of 03:00 and 06:00 revealed PET values of − 29.3 °C and − 30.0 °C, respectively. For the same period, diurnal measurements at 12:00 and 15:00 continued to reveal a PS vulnerability of CS6, thus inferring the unremitting susceptibility to such risk factors for both January and February. Interestingly, although the 2012 dataset revealed the coldest winter conditions (Fig. 5), events of extreme HS were still acknowledged during the summer, even in the cooler peri-urban region of Ankara.

Adjacently, representing one of the hottest yearly datasets, 2010 demonstrated both the vulnerability to extreme heatwaves during July and August which sometimes consecutively remained between HS3 and HS5 for longer than a week with maximum PET values reaching 48 °C (Fig. 6b**)**. Irrespective to this hotter dataset, it was nevertheless still possible to additionally identify periods of extreme CS during the winter at the end of January, again inferring the considerable variability of annual PS levels in city centre as well.

#### Frequency distribution analysis

The FDDs depicted the resulting probability to PS grades for the nocturnal and diurnal period. Moreover, it was possible to equate the variances both between the MSs and the two EBM indices.

The first FDD results demonstrated the probability of PS at 03:00 which denoted the coldest measurement hour. The synoptically expanded thermophysiological levels as discussed in Table 2 enabled PS variations to be further classified PET/mPET values below 4 °C. Given the obtained results, particularly in the case of the PET, the original lower CS limit (equating to the CS3) would not have been able to effectively plot the more extreme climatic conditions acknowledged for the months between November and April. In the particular case of the peri-urban MS, and remembering that the FDDs were based upon yearly dataset averages, for the first 10 days in February, it was possible to identify a (i) CS7 probability of 5%, (ii) CS6 probability of 33%, (iii) CS5 probability of 59%, and (iv) CS4 probability of 3%. In comparison, mPET results revealed lower probability of extreme CS, and the overall likelihood of CS3 was much higher.

**Fig. 7:**
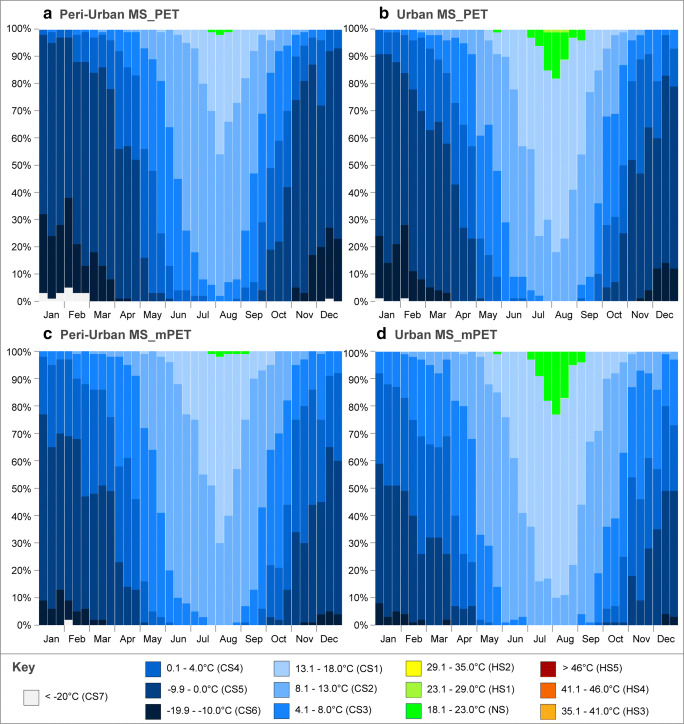
Frequency distribution diagram of PS grades for PET and mPET at 03:00 peri-urban MS and urban MS. **a** Peri-urban MS_PET. **b** Urban MS_PET. **c** Peri-urban MS_mPET. **d** Urban MS_mPET

When considering the results presented in Fig. 7 b, d, the increased vulnerability to hotter environmental conditions for the urban MS was as again confirmed as a result of the lower probability of CS, and higher likelihood of NS, which during early August surpassed 20% in the case of mPET. In addition, the probability of CS1 was much higher for both PET and mPET.

**Fig. 8:**
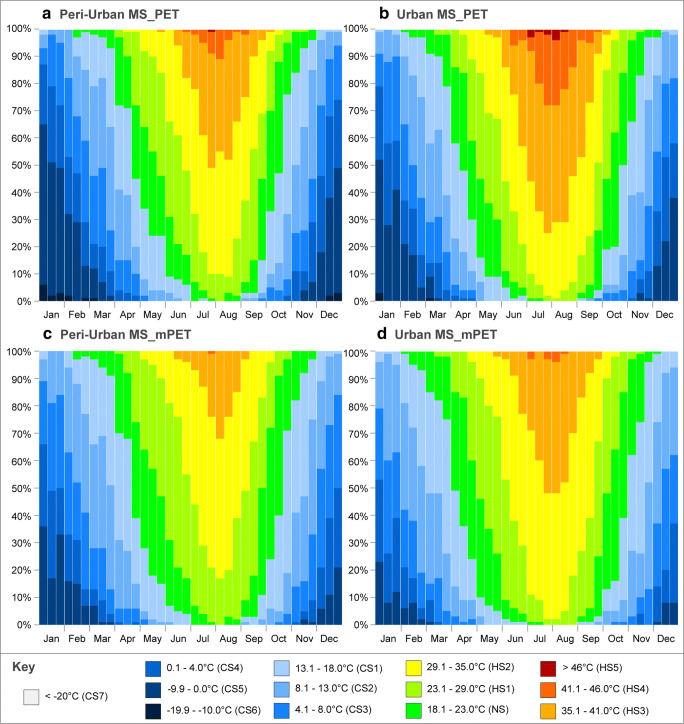
Frequency distribution diagram of PS grades for PET and mPET at 15:00 for the peri-urban MS and urban MS. **a** Peri-urban MS_PET. **b** Urban MS_PET. **c** Peri-urban MS_mPET. **d** Urban MS_mPET

In the case of hottest measurement hour, 15:00 naturally rendered very different PS frequency percentages within Fig. 8. Nevertheless, the recurring patterns between the PET and mPET indices and amongst the urban and peri-urban MSs continued. In both the MSs, the mPET revealed very low probability of HS4 during the summer months and most often remained at HS3. Notwithstanding, in the case of the peri-urban MS, even HS3 frequency was considerably lower, reaching 32% in early August, as opposed to 49% in the case of the urban MS (Fig. 8c, d).

In the case of PET, PS levels reached HS5 in the city centre with a probability of 3/4% in early July and August. Although a more negligible likelihood, it was important to remember once again that the FDDs were based upon the average EBM values of all yearly datasets, hence indicating higher probabilities within the individual years. Irrespectively, and even through the average frequency analysis over the past decade, Fig. 8b revealed considerable risks for human health within Ankara’s city centre, which as exemplified for early August witnessed a (i) HS5 probability of 4%, (ii) HS4 probability of 24%, (iii) HS3 probability of 43%, and (iv) HS2 probability of 28%.

#### Decadal average maximum and minimum values

**Fig. 9:**
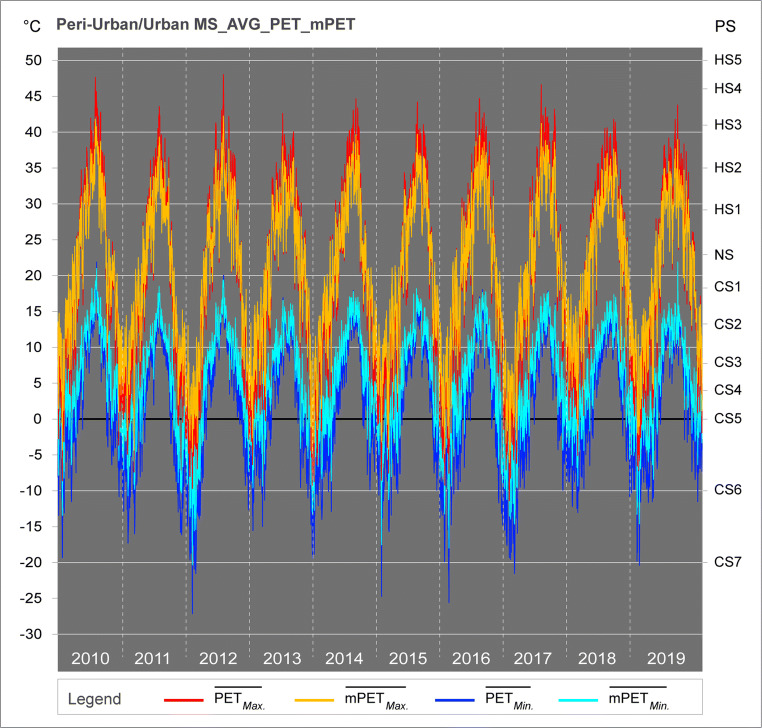
Average yearly variability of Min. and Max. values of PET and mPET for peri-urban/urban MSs

The EBM analysis also permitted to verify if there were any general trends between the yearly datasets and moreover the respective m/PET averages of $\overline{\operatorname{Min}.}$ and $\overline{\operatorname{Max}.}$ values for the two MSs. The results demonstrated in Fig. 9 revealed that while there were no overall incremental/decremental trends in the past decade, it was possible to recognise the high variability of both EBM indices between 2011 and 2019. As an example, the 2012 dataset in particular demonstrated the greatest variability between the annual $\overline{{\mathrm{PET}}_{\operatorname{Min}.}}$ of − 27.2 °C and $\overline{{\mathrm{PET}}_{\operatorname{Max}.}}$ of 48.1 °C, which in numerical terms, corresponds to a variation of 75 °C between the summer and the winter months. The 2010 dataset presented the second highest $\overline{{\mathrm{PET}}_{\operatorname{Max}.}}$ of 48.1 °C. Although based on average values from both MSs, the influences of such extreme conditions during 2010 and 2012 can be directly correlated with the results presented in Figs. 5 and 6. In the case of mPET, the index also followed such trends, yet did not reach such extreme thermophysiological values.

## Discussion

### Behavioural differences between the EBMs

The results of the study identified clear behavioural differences between the three processed EBM indices. Moreover, and comparing such results to the existing literature, the results obtained for the case of Ankara highlighted the (i) tendency for PET to fluctuate further from NS levels in comparison to mPET due to the expansion of the integrated thermoregulation and clothing models, particularly under extreme environmental conditions (in similarity with, e.g., Chen and Matzarakis 2017; Lin et al. 2018; Nouri et al. 2017); (ii) importance to consider adaptations of the original PS thresholds associated to the MEMI indices (in similarity with, e.g., Chen et al. 2020; Hwang and Lin 2007; Matzarakis 2014a; Potchter et al. 2018), which in turn, calls for further study to better delineate the relationship of such expanded HS and CS levels upon the human biometeorological system (in accordance with, e.g., Nouri et al. 2018a; Nouri et al. 2018c); (iii) opportunity for further study regarding the more recent mPET index in light of its adaptations from the original MEMI (Staiger et al. 2019), including also its relationship with the utilised PS grade system as originally defined by Matzarakis et al. (1999); (iv) problematical relationship between the two MEMI indices with the Fiala model-based UTCI due their different algorithmic calculation methods, and differing association with designated PS levels.

Discussing such results between the MEMI indices in a little more detail, during the winter period, nocturnal PET always revealed higher probabilities of CS in comparison to mPET. As exemplified for the peri-urban MS, while winter months revealed higher PET PS probabilities of CS7 (relaying to temperatures < − 20 °C), CS6, and CS5 (with a cumulative probability almost 100% as exemplified in early February); mPET on the other hand, presented generally more attenuated stress levels, with a greater likelihood of CS5 and CS4 (with a cumulative probability of almost 80% for the same period). Illustrated by the urban MS, similar patterns were also identified for the nocturnal hours during the summer period, where mPET were more conducive of PS grades closer to NS levels, particularly in the case of NS, CS1, and CS2. During the summer period, and at the urban MS, diurnal mPET continued to show lower probabilities of higher PS levels, revealing a limited likelihood of HS4, reaching a maximum of 4%, and a subsequent HS3 probability of 48%. These results were sharply divergent from PET results, with a maximum HS5 (relaying to temperatures above > 46 °C) probability of 4%, and a following HS4 probability of 26%.

With regard to the UTCI index, its calculation was more complex in that it was also hindered as a result of the frequent environmental circumstances that witnessed local low V_1.1_ speeds, hence limiting its estimation in short temporal periods that were not composed of average values. Even in such average assessments, the disclosed hindrances were clear as a result of the more ‘choppy’ outputs as delineated by the undertaken CTIS analysis. In addition, the more ‘versatile’ adaptive clothing model as presented by Havenith et al. (2012) must be approached with caution due to the model being predominantly based upon European dressing behaviour/standards. As stated in the results section, although the comparison of UTCI with mPET and PET is innately equivocal, some similarities between the two MEMI indices were identified, particularly with PET during hotter PS conditions. In addition, and concomitant with PET and mPET, the UTCI index also confirmed the same general environmental differences for Ankara’s urban and peri-urban regions.

### Variation of thermophysiological conditions between stations

Building upon the line of reasoning as aforementioned in the previous section, the study was able to identify very clear thermophysiological conditions over the past decade between the peri-urban and urban MSs. Located ~ 20 km away from one another, it was possible to acknowledge the clear influences as a result of their different contexts. These outcomes confirmed (with regard to PET in particular) the previous results as identified by Türkoğlu et al. (2012) and Çalışkan and Türkoğlu (2014) for the case of Ankara.

As also initially indicated on behalf of the singular variables, it was possible to identify numerous clear differences amid the MSs, including higher V_1.1_ values in the peri-urban MS, and T_a_/T_mrt_ in the urban MS. In the case of V_1.1_, the season with the greatest variations was during the spring, with $\overline{\mathrm{Mean}.}$ and $\overline{\operatorname{Max}.}$ values remaining constantly higher in the peri-urban MS, with differences reaching 1.1 K and 4.0 K, respectively.

Overall, while the peri-urban MS revealed an annual $\overline{\mathrm{Mean}.}$ T_a_ of 11.2 °C, such a value increase to 13.3 °C in the case of the urban MS. In terms of highest $\overline{\operatorname{Max}.}$ and $\overline{\operatorname{Min}.}$ vacillations, it was possible to ascertain a (i) T_a_ variation of 4.2 K in $\overline{\operatorname{Max}.}$ values during the summer, with the hotter temperatures being recorded at the urban MS at 21:00; and (ii) T_a_ variation of 7.5 K in $\overline{\operatorname{Min}.}$ values during the winter, with the colder temperatures being retrieved from the peri-urban MS at 03:00. With regard to T_mrt_, it was possible to identify that while the urban MS largely revealed the highest values, such disparities were particularly manifested in the $\overline{\operatorname{Min}.}$ seasonal values, as exemplified by a difference of 13.3 K and 18.9 K during the summer and winter seasons, respectively.

Given the considerably higher thermophysiological conditions at the urban MS, such results had direct impacts upon the wholesome EBM indices, and were clear indications of the bearings of UHI intensities and effects within consolidated city centres; such influences moreover link with the outcomes (and potential mitigation strategies) as identified in analogous international studies (e.g., Alcoforado and Andrade 2006; Alcoforado et al. 2014; Cheval and Dumitrescu 2008; Dimoudi et al. 2014; Lopes et al. 2013; Matzarakis et al. 2016; Santamouris 2013; Wang et al. 2016). In the case of Ankara, further study is hence required to (i) further comprehend the specific particularities of UHI intensities and vulnerabilities within the city centre, and just as importantly, (ii) to consider local urban design/planning approaches to further improve the urban centre response to such adverse environmental effects.

### Overall PS conditions over the last decade

When considering the overall thermophysiological conditions for Ankara during the last decade, it was possible to identify a wide range of PS levels as a result of the significant variations in the processed EBM indices. Consequently, the results presented by the two MSs revealed different types of human biometeorological risk factors during the winter and summer months. Such acknowledged thermal comfort hazards are resultant of three interconnected environmental factors associated to the ‘human-centred approach’, these being the (i) extreme hourly and/or daily EBM index values that rendered high HS or CS levels during the different seasons; (ii) lower HS and CS levels, which while not always concomitant with extreme PS classifications, still presented serious implications upon cumulative human thermal stress as a result of their continuous circadian frequency; and lastly, (iii) the combination of both extended periods of PS and the additional occurrence of extreme climatic events which further exacerbated each of the respective human thermal risk factors.

## Concluding remarks

Within the existing literature, approaches towards human thermophysiological conditions and their associated biometeorological risk factors are continuing to mature in the scope of urban environmental studies. Concomitant to such a continual scientific development is the consolidating ‘human-centred approach’ that is focused upon understanding the direct effects of microclimatic stimulus upon the human body, and how these dynamics can be locally identified. Within this particular study, both the peri-urban and urban MSs revealed different environmental conditions both in terms of singular variables and EBM indices.

In the case of T_a_, the higher temperatures found in Ankara’s city centre which varied up to 4.2 K during the summer season, and 7.5 K for the winter, revealed considerable local disparities. Nevertheless, in biometeorological terms, to obtain a wholesome understanding of such environmental stimulus on the human body, it was also necessary to cumulatively consider the additional implications, namely the (i) lower urban wind velocities that would otherwise serve to cool the public realm through net heat advection; (ii) the often marginally higher general VP values in the urban centre during the summer which increased the risk of dehydration due to higher saturation deficits; (iii) predominantly higher T_mrt_ values resulting from the urban MS which a maximum difference of 13.3 K recorded at 15:00 during the summer months, thus indicating the higher radiant heat exchange rates with the human body, further exacerbating the thermal stimulus upon the biometeorological system.

The calculated EBM indices were able to present a wholesome evaluation of all of the aforementioned environmental impacts upon the human body. In the case of UTCI, while it was able to reveal some similarities to the MEMI indices, its outcomes were more partial in light of its (i) hindrances associated to lower V_1.1_ conditions, and (ii) enclosed clothing model that is based upon European clothing patterns. Nevertheless, all EBMs were able to recognise that the city centre presented hotter environmental conditions in comparison to those registered by the peri-urban station. Although resulting in more attenuated levels of CS during the winter, summer HS was frequently exacerbated, both in terms of recognised extreme values, and also, in the temporal continuity of such augmented PS levels.

In addition, and concomitant to the previous result, all of the EBM indices revealed clear PS oscillations between the nocturnal measurements. The acknowledged variations were further highlighted with 03:00 revealing to be the coldest measurement hour, and 15:00 frequently being the hottest during the year. Between these two bounds, PS levels also oscillated accordingly in light of the yearly sunset/sunrise times. As to be expected, and in light of the lack of radiant heat exchange, the nocturnal periods resulted in much cooler conditions even when PS reached HS4 or HS5 during mid-day. Naturally, the reverse effect during the winter was also valid, in that diurnal PS levels were slightly more attenuated, particularly in the case of mPET. In juxtaposition, and particularly during periods of augmented thermophysiological stimulus, mPET recurrently presented more attenuated PS levels in comparison to PET extremes which were able to vary by 75 °C in the same dataset. Given such fluctuating environmental human thermophysiological conditions for Ankara, it was not suitable to associate PS risk factors only to events such as urban extreme heatwaves or very hot days during the summer months.

Under a more general perspective, the thermal indices scrutinised in this article demonstrated how the case study of Ankara highlighted not only the importance of the ‘human-centred approach’, but in addition, the relevance of the EBM indices as well. Although parting from different calculation methods (i.e., the Fiala and MEMI methodologies), the three different EBM indices revealed how the context of Ankara further extrapolated individual performances under different climatic settings, and also, how they are presented as wholesome information based upon singular climatic variables.

Respectively, the context and climatic typology of Ankara is an important case study due to its vast amplitudes in thermophysiological variations and frequencies amongst all EBMs. Moreover, and within this line of reasoning, serves to further knowhow upon similar settings, namely general climatic conditions (e.g., those associated to KG ‘*BSk*’ or ‘*Dsb*/*a*’ classifications), geographical location, and local/encircling topographical configurations). As identified, the differences between UTCI, mPET, and PET should not be compared in light of their presented PS levels, but rather, how they produced different biometeorological results, information on thermal comfort thresholds, and just as significantly, their limitations (more correctly labelled as opportunity for further development). In addition, the vast differences in thermophysiological thresholds provided a fertile demesne in which to test their performance in both cold and hot extremes identified in Ankara’s encompassing climatic conditions—both in its urban and peri-urban settings. Such extensive variations also enabled the study to build upon uprising and initial approaches in extend existing PS grades in the case of PET and mPET. Such an approach particularly enabled a better identification of maximum CS extensions during the winter, as exemplified by the 2012 dataset. Naturally, and as mentioned, such extended grades should be seen simultaneously with the opportunity to undertake further study, particularly with regard to their labelling given their direct biometeorological implications upon the human body.

Within the study, and going beyond the decadal environmental condition analysis, the benefits and limitations between each of the EBM indices could be looked into detail, during all seasons, and both diurnal/nocturnal periods. The fluctuation tendencies in extreme conditions between PET and mPET from NS levels were clear due to the expansion of the integrated thermoregulation and clothing models. While this suggested the potential limitation of PET in this sense, it adjacently raised the need for further study for the more recent mPET index that deviates from the original MEMI approach. In the case of UTCI, while it also performed effectively in donating the inferences of wholesome evaluations of singular variables upon the human body, its issue with lower wind speeds hindered its calculation in short temporal periods that were not composed of average values. Even with such averages, the results were not as complete within the undertaken CTIS analysis in this study. Resultantly, further study is required with regard to this EBM index in terms, including also the adaptive clothing model, which while adaptive, remains based upon the European clothing standards and behaviour.

Categorically, none of the aforementioned requirements for further study should be considered to negate, by any means, the existing capacity of any of the EBMs for application and utilisation for these types of biometeorological approaches. On the contrary, based upon the results presented in this paper, it is suggested that each provide clear benefits for thermal comfort evaluations, including for non-climatic experts such as architects and urban planners/designers to acknowledge and explore concrete means to tackle such human thermophysiological thresholds within urban contexts through a wholesome thermophysiological ‘human-centred approach’. Furthermore, it is also suggested that this issue shall become continually more crucial and sought after in an era that is already witnessing the predicted aggravation effects associated to climate change.

## Abbreviations

CS#: Cold stress (level 1–7)
CTIS: Climate-Tourism/Transfer-Information-Scheme
DJF: December January February
EBM: Energy balance model
FDDs: Frequency distribution diagrams
GCMs: Global circulation models
HS#: Heat stress (level 1–7)
JJA: June July August
KG: Köppen-Geiger
MAM: March April May
MEMI: Munich energy-balance model for individuals
mPET: Modified physiologically equivalent temperature (°C)
MSs: Meteorological stations
NS: No thermal stress
Oct: Cloud cover
PET: Physiologically equivalent temperature (°C)
P_R_: Precipitation
PR: Perspiration rate
PS: Physiological stress
PT: Perceived temperature (°C)
RCMs: Regional climate models
RH: Relative humidity (%)
SET*: Standard effective temperature (°C)
SON: September October November
T_a_: Air temperature (°C)
T_Cr_: Core temperature (°C)
T_mrt_: Mean radiant temperature (°C)
TÜBİTAK: Scientific and Technological Research Council of Turkey
T_Sk_: Skin temperature (°C)
UHI: Urban heat island
UTCI: Universal thermal climate index (°C)
V: Wind speed (m/s)
V_1.1_: Wind speed at 1.1 m from ground (m/s)
VP: Vapour pressure (hPA)

## References

[CR1] Alcoforado MJ (1996). Comparasion des ambiances bioclimatiques estivales d’espaces verts de Lisbonne. J Assoc Interationale Climatol.

[CR2] Alcoforado MJ, Andrade H (2006). Nocturnal urban heat island in Lisbon (Portugal): main features and modelling attempts. Theor Appl Climatol.

[CR3] Alcoforado MJ, Andrade H (2007) Microclimatic variation of thermal comfort in a district of Lisbon (Telheiras) at night. Theor Appl Climatol 13. 10.1007/s00704-007-0321-5

[CR4] Alcoforado MJ, Lopes A, Alves E, Canário P (2014). Lisbon heat island statistical study. Finisterra.

[CR5] Algeciras JAR, Matzarakis A (2015). Quantification of thermal bioclimate for the management of urban design in Mediterranean climate of Barcelona, Spain. Int J Biometeorol.

[CR6] Andreou E (2013). Thermal comfort in outdoor spaces and urban canyon microclimate. Renew Energy.

[CR7] Binarti F, Koerniawan M, Triyadi S, Utami S, Matzarakis A (2020). A review of outdoor thermal comfort indices and neutral ranges for hot-humid regions. Urban Clim.

[CR8] Brebner D, Kerslake D, Waddell J (1958). The effect of atmospheric humidity on the skin temperatures and sweat rates of resting men at two ambient temperatures. J Physiol.

[CR9] Bröde P (2012). Deriving the operational procedure for the universal thermal climate index (UTCI). Int J Biometeorol.

[CR10] Çalışkan O, Türkoğlu N (2014). The trends and effects of urbanization on thermal comfort conditions in Ankara. Coğrafi Bilimler Dergisi.

[CR11] Çalışkan O, Çiçek İ, Matzarakis A (2012). The climate and bioclimate of Bursa (Turkey) from the perspective of tourism. Theor Appl Climatol.

[CR12] Charalampopoulos I (2019). A comparative sensitivity analysis of human thermal comfort indices with generalized additive models. Theor Appl Climatol.

[CR13] Charalampopoulos I, Nouri AS (2019). Investigating the behaviour of human thermal indices under divergent atmospheric conditions: a sensitivity analysis approach. Atmosphere.

[CR14] Charalampopoulos I, Tsiros I, Chronopoulou-Sereli A, Matzarakis A (2016). A methodology for the evaluation for the human-bioclimatic performance of open spaces. Theor Appl Climatol.

[CR15] Chen D, Chen HW (2013). Using the Köppen classification to quantify climate variation and change: an example for 1901-2010. J Environ Dev.

[CR16] Chen Y-C, Matzarakis A (2017). Modified physiologically equivalent temperature - basics and applications for western European climate. Theor Appl Climatol.

[CR17] Chen Y-C, Chen W, Chou C, Matzarakis A (2020). Concepts and new implements for modified physiologically equivalent temperature. Atmosphere.

[CR18] Cheval S, Dumitrescu A (2008). The Julu urban heat island of Bucharest as derived from MODIS images. Theor Appl Climatol.

[CR19] Cheval S, Adamescu C, Georgiadis T, Herrengger M, Piticar A, Legates D (2020). Observed and potential impacts of the COVID-19 pandemic on the environment. Int J Environ Res Public Health.

[CR20] Christen A, Christen A, Matzarakis A (2020). Future perspectives at the intersection between urban climate and human biometeorology. Symposium on challenges in applied human biometeorology.

[CR21] Coccolo S, Kämpf J, Scartezzini J, Pearlmutter D (2016). Outdoor thermal comfort and thermal stress: a comprehensive review on models and standards. Urban Clim.

[CR22] de-Dear R, Brager G (1998). Developing an adaptative model of thermal comfort preference. ASHRAE Trans.

[CR23] Dimoudi A, Zoras S, Kantzioura A, Stogiannou X, Kosmopoulos P, Pallas C (2014). Use of cool materials and other bioclimatic interventions in outdoor places in order to mitigate the urban heat island in a medium size city in Greece. Sustain Cities Soc.

[CR24] Djamila H, Yong T (2016). A study of Köppen-Geiger system for comfort temperature prediction in Melbourne city. Sustain Cities Soc.

[CR25] Fiala D, Havenith G, Bröde P, Kampmann B, Jendritzky G (2012). UTCI-Fiala multi-node model of human heat transfer and temperature regulation. Int J Biometeorol.

[CR26] Freitas C, Grigorieva E (2015). A comprehensive catalogue and classification of human thermal climate indices. Int J Biometeorol.

[CR27] Freitas C, Grigorieva E (2016). A comparison and appraisal of a comprehensive range of human thermal climate indices. Int J Biometeorol.

[CR28] Fröhlich D, Gangwisch M, Matzarakis A (2019). Effect of radiation and wind on thermal comfort in urban environments - applications of the RayMan and SkyHelios. Model Urban Climate.

[CR29] Gagge A, Fobelets P, Bergland L (1986). A standard predictive index of human response to thermal environment. ASHRAE Trans.

[CR30] Giannaros T, Lagouvardos K, Kotroni V, Matzarakis A (2018). Operational forecasting of human-biometeorological conditions. Int J Biometeorol.

[CR31] Gonzalez R, Nishi Y, Gagge A (1974). Experimental evaluation of standard effective temperature: a new biometeorological index of man’s thermal discomfort. Int J Biometeorol.

[CR32] Havenith G, Fiala D, Błazejczyk K, Richards M, Bröde P, Holmér I, Rintamaki H, Benshabat Y, Jendritzky G (2012). The UTCI-clothing model. Int J Biometeorol.

[CR33] Hebbert M, Mackillop F (2011). Urban climatology applied to urban planning: A postwar knowledge circulation failure. Int J Urban Reg Res.

[CR34] Hebbert M, Webb B (2007). Towards a liveable urban climate: lessons from Stuttgart. Liveable cities: Urbanising World: Isocarp 07.

[CR35] Hensel H, Schafer K, Ring E, Philips B (1984). Thermoreception and temperature, regulation in man. Recent advances in medical thermology.

[CR36] Herrmann J, Matzarakis A (2012). Mean radiant temperature in idealised urban canyons - examples from Freiburg, Germany. Int J Biometeorol.

[CR37] Holmer B, Eliasson I (1999). Urban-rural vapour pressure differences and their role in the development of urban heat islands. Int J Climatol.

[CR38] Holopainen R (2012) A human thermal model for improved thermal comfort. Aalto University

[CR39] Höppe P (1984). The energy balance in humans (Original title - Die Energiebilanz des menschen) Wissenschaftliche Mitteilungen.

[CR40] Höppe P (1993). Heat balance modelling. Experientia.

[CR41] Höppe P (1999). The physiological equivalent temperature - a universal index for the biometeorological assessment of the thermal environment. Int J Biometeorol.

[CR42] Hwang L, Lin T-P (2007). Thermal comfort requirements for occupants of semi-outdoor and outdoor environments in hot-humid regions. J Archit Sci Rev.

[CR43] Hwang R-L, Lin T-P, Matzarakis A (2010). Seasonal effects of urban street shading on long-term outdoor thermal comfort. Build Environ.

[CR44] Jendritzky G, Maarouf A, Fiala D, Staiger H (2002) An update on the development of a universal thermal climate index. Paper presented at the 15th Conf. Biomet. Aerobiol. and 16th ICB02 Kansas City

[CR45] Jendritzky G, de-Dear R, Havenith G (2012). UTCI - why another thermal index?. Int J Biometeorol.

[CR46] Kántor N, Chen L, Gal CV (2018). Human-biometeorological significance of shading in urban public spaces—summertime measurements in Pécs, Hungary. Landsc Urban Plan.

[CR47] Katić K, Li R, Zeiler W (2016). Thermophysiological models and their applications: a review. Build Environ.

[CR48] Ketterer C, Matzarakis A (2014). Human-biometeorological assessment of heat stress reduction by replanning measures in Stuttgart, Germany. Landsc Urban Plan.

[CR49] Kuttler W (2000) Stadtklima. In: Guderian R (ed) Handbuch der Umweltveränderungen und Ökotoxologie, Band 1B:Springer, Berlin, p 470

[CR50] Lin T-P (2009). Thermal perception, adaptation and attendance in a public square in hot and humid regions. Build Environ.

[CR51] Lin T-P, Matzarakis A (2008). Tourism climate and thermal comfort in Sun Moon Lake, Taiwan. Int J Biometeorol.

[CR52] Lin T-P, Yang S-R, Matzarakis A (2015). Customized rating assessment of climate suitability (CRACS); climate satisfaction evaluation based on subjective perception. Int J Biometeorol.

[CR53] Lin T-P, Yang S-R, Chen Y-C, Matzarakis A (2018). The potential of a modified physiologically equivalent temperature (mPET) based on local thermal comfort perception in hot and humid regions. Theor Appl Climatol.

[CR54] Lopes A, Alves E, Alcoforado MJ, Machete R (2013). Lisbon Urban Heat Island updated: new highlights about the relationships between thermal patterns and wind regimes. J Adv Meteorol.

[CR55] Matzarakis A, Matzarakis A, Freitas C, Scott D (2007). Climate and bioclimate information for tourism - the example of Evros prefecture. Developments in tourism climatology.

[CR56] Matzarakis A (2014a) Aufbereitung und Analyse von Klimawandeldaten für den Tourismus – Das Klima-Tourismus/Transfer-Informations-Schema (CTIS). In: Strasdas W, Zeppenfeld R (eds) Tourismus und Klimawandel in Mitteleuropa. Wissenschaft trifft Praxis – Ergebnisse der Potsdamer Konferenz, vol 2014. Springer, Wiesbaden, pp 39–49

[CR57] Matzarakis A (2014). Transfer of climate data for tourism applications - the climate-tourism/transfer-information-scheme. J Environ Res.

[CR58] Matzarakis A, Amelung B, Thomson MC, Garcia-Herrera R, Beniston M (2008). Physiological equivalent temperature as indicator for impacts of climate change on thermal comfort of humans. Seasonal forecasts, climatic change and human health. Advances in global research 30.

[CR59] Matzarakis A, Karagülle M, Matzarakis A, Freitas C, Scott D (2007). Bioclimate information for Istanbul. Developments in tourism climatology.

[CR60] Matzarakis A, Mayer H (1997). Heat stress in Greece. Int J Biometeorol.

[CR61] Matzarakis A, Rutz F (2006) Rayman Pro - modelling of mean radiant temperature in urban structures calculation of thermal indices. vol Version 2.1 Pro. Meteorological institute, University of Freiburg, Germany

[CR62] Matzarakis A, Mayer H, Iziomon GM (1999). Applications of a universal thermal index: physiological equivalent temperature. Int J Biometeorol.

[CR63] Matzarakis A, Rutz F, Mayer H (2010). Modelling radiation fluxes in simple and complex environments - basics of the RayMan model. Int J Biometeorol.

[CR64] Matzarakis A, Fröhlich D, Gangwisch M (2016) Effect of radiation and wind on thermal comfort in urban environments - applications of the RayMan and SkyHelios model. Paper presented at the 4th International Conference on Countermeasures to Urban Heat Island, National University of Singapore, Singapore

[CR65] Mayer H, Höppe P (1987). Thermal comfort of man in different urban environments. Theor Appl Climatol.

[CR66] Miao C, Yu S, Hu Y, Zhang H, He X, Chen W (2019). Review of methods used to estimate the sky view factor in urban street canyons. Build Environ.

[CR67] Mole R (1948). The relative humidity of the skin. J Physiol.

[CR68] Nicol F (2004). Adaptive thermal comfort standards in the hot-humid tropics. Energ Build.

[CR69] Nouri AS, Bártolo H (2013). A bottom-up perspective upon climate change - approaches towards the local scale and microclimatic assessment. Green design, materials and manufacturing processes.

[CR70] Nouri AS, Costa JP (2017). Addressing thermophysiological thresholds and psychological aspects during hot and dry Mediterranean summers through public space design: the case of Rossio. Build Environ.

[CR71] Nouri AS, Matzarakis A (2019). The maturing interdisciplinary relationship between human biometeorological aspects and local adaptation processes: an encompassing overview. Climate.

[CR72] Nouri AS, Matzarakis A (2020) Human biometeorological models - existing and future reflections for Lisbon. In: Palme M, Salvati A (eds) Urban microclimatic simulation for comfort and energy studies. Springer Nature Switzerland, pp 1–19 (In Press)

[CR73] Nouri AS, Costa JP, Matzarakis A (2017). Examining default urban-aspect-ratios and sky-view-factors to identify priorities for thermal-sensitive public space design in hot-summer Mediterranean climates: the Lisbon case. Build Environ.

[CR74] Nouri AS, Charalampopoulos I, Matzarakis A (2018). Beyond singular climatic variables - identifying the dynamics of wholesome thermo-physiological factors for existing/future human thermal comfort during hot dry Mediterranean summers. Int J Environ Res Public Health.

[CR75] Nouri AS, Fröhlich D, Silva MM, Matzarakis A (2018). The impact of Tipuana tipu species on local human thermal comfort thresholds in different urban canyon cases in Mediterranean climates: Lisbon, Portugal. Atmosphere.

[CR76] Nouri AS, Lopes A, Costa JP, Matzarakis A (2018). Confronting potential future augmentations of the physiologically equivalent temperature through public space design: the case of Rossio, Lisbon. Sustain Cities Soc.

[CR77] Oke T (1988). The urban energy balance journal of. Prog Phys Geogr.

[CR78] Olgyay V (1963). Design with climate, bioclimatic approach to architectural regionalism.

[CR79] Öztürk M, Çetinkaya G, Aydin S (2017). Climate types of Turkey according to Köppen-Geiger climate classification. J Geogr.

[CR80] Parsons K (2003). The effects of hot, moderate, and cold environments on human health, comfort and performance.

[CR81] Peel M, Finlayson B, McMahon T (2007). Updated world map of the Koppen-Geiger climate classification. J Hydrol Earth Syst Sci.

[CR82] Potchter O, Cohen P, Lin T, Matzarakis A (2018). Outdoor human thermal perception in various climates: a comprehensive review of approaches, methods and quantification. Sci Total Environ.

[CR83] Provençal S, Bergeron O, Leduc R, Barrette N (2016). Thermal comfort in Quebec City, Canada: sensitivity analysis of the UTCI and other popular thermal comfort indices in a mid-latitude continental city. Int J Biometeorol.

[CR84] Reiter S, Herde Ad (2003) Qualitative and quantitative criteria for comfortable urban public spaces. ORBi, Proceedings of the 2nd International Conference on Building Physics

[CR85] Rubel F, Kottek M (2010). Observed and projected climate shifts 1901-2100 depicted by world maps of the Köppen-Geiger climate classification. J Meteorol Zeitschrift.

[CR86] Rubel F, Kottek M (2011). Comments on: “The thermal zones of the Earth” by Wladimir Köppen (1884). Meteorol Z.

[CR87] Santamouris M (2013). Using cool pavements as a mitigation strategy to fight urban heat islands - a review of the actual developments. J Renew Sust Energ Rev.

[CR88] Şensoy S, Türkoğlu N, Çiçek İ, Matzarakis A (2020). Thermal comfort features of Antalya, future projections using climate model data and its effects on tourism Turkish. J Geogr Sci.

[CR89] Shashua-Bar L, Tsiros IX, Hoffman M (2012). Passive cooling design options to ameliorate thermal comfort in urban streets of a Mediterranean climate (Athens) under hot summer conditions. Build Environ.

[CR90] Spagnolo J, de-Dear R (2003). A field study of thermal comfort in outdoor and semi-outdoor environments in subtropical Sydney, Australia. Build Environ.

[CR91] Staiger H, Laschewski G, Gratz A (2012). The perceived temperature - a versatile index for the assessment of the human thermal environment. Part A: scientific basics. Int J Biometeorol.

[CR92] Staiger H, Laschewski G, Matzarakis A (2019). Selection of appropriate thermal indices for applications in human biometeorological studies. Atmosphere.

[CR93] Thorsson S, Rocklöv J, Konarska J, Lindberg F, Holmer B, Dousset B, Rayner D (2014). Mean radiant temperature - a predictor of heat related mortality. Urban Clim.

[CR94] Thorsson S (2017). Present and projected future mean radiant temperature for three European cities. Int J Biometeorol.

[CR95] Türkoğlu N, Çalışkan O, Çiçek İ, Yılmaz E (2012). The analysis of impact of urbanization on the bioclimatic conditions in the scale of Ankara. Uluslararası Insan Bilimleri Dergisi.

[CR96] Unal Y, Kindap T, Karaca M (2003). Redefining the climate zones of Turkey using cluster analysis. Int J Climatol.

[CR97] Vanos J, Warland J, Gillespie T, Kenny N (2010). Review of the physiology of human thermal comfort while exercising in urban landscapes and implications for bioclimatic design. Int J Biometeorol.

[CR98] VDI (1998) Part I: environmental meteorology, methods for the human-biometeorological evaluation of climate and air quality for the urban and regional planning at regional level. Part I: climate. VDI/DIN-Handbuch Reinhaltung der Luft. Verein Deutscher Ingenieure, Düsseldorf, Germany

[CR99] Walton D, Dravitzki V, Donn M (2007). The relative influence of wind, sunlight and temperatures on used comfort in urban outdoor spaces. Build Environ.

[CR100] Wang Y, Berardi U, Akbari H (2016). Comparing the effects of urban heat island mitigation strategies for Toronto, Canada. Energ Build.

[CR101] Wilbanks TJ, Kates RW (1999) Global change in local places: how scale matters vol 43. The Netherlands

[CR102] Wu Y, Graw K, Matzarakis A (2020). Comparison of thermal comfort between Sapporo and Tokyo - the case of the Olympics 2020. Atmosphere.

[CR103] Yang S, Matzarakis A (2016). Implementation of human thermal comfort information in Köppen-Geiger climate classification - the example of China. Int J Biometeorol.

[CR104] Yang S, Matzarakis A (2019). Implementation of human thermal comfort and air humidity in Köppen-Geiger climate classification and importance towards the achievement of sustainable development goals. Theor Appl Climatol.

[CR105] Yılmaz E, Çiçek İ (2018). Detailed Köppen-Geiger climate regions of Turkey. Int J Hum Sci.

[CR106] Yilmaz S, Irmak M, Matzarakis A (2013). The importance of thermal comfort in different elevation for city planning. Global NEST J.

[CR107] Zare S, Hasheminejad N, Shirvan H, Hemmatjo R, Sarebanzadeh K, Ahmadi S (2018). Comparing universal thermal climate index (UTCI) with selected thermal indices/environmental parameters during 12 months of the year. Weath Clim Extremes.

